# Quercetin in Tartary Buckwheat Induces Autophagy against Protein Aggregations

**DOI:** 10.3390/antiox10081217

**Published:** 2021-07-29

**Authors:** Sumiko Ikari, Qiang Yang, Shiou-Ling Lu, Yuancai Liu, Feike Hao, Guoqiang Tong, Shiguang Lu, Takeshi Noda

**Affiliations:** 1 Center for Frontier of Oral Sciences, Graduate School of Dentistry, Osaka University, Osaka 5650871, Japan; sikari@kuhp.kyoto-u.ac.jp (S.I.); sally711017@dent.osaka-u.ac.jp (S.-L.L.); haofeike@outlook.com (F.H.); 2 Hubei Provincial Key Lab for Quality and Safety of Traditional Chinese Medicine Health Food, Daye 435100, China; yq@jingpai.com (Q.Y.); lyc@jingpai.com (Y.L.); 3 Jing Brand Company, Ltd., Daye 435100, China; winebag@jingpai.com (G.T.); lushiguang@jingpai.com (S.L.)

**Keywords:** tartary buckwheat, mTORC1, aggrephagy

## Abstract

Tartary buckwheat is used as an ingredient in flour and tea, as well as in traditional Chinese medicine for its antioxidant effects. Here, we found that an ethanol extract of tartary buckwheat (TBE) potently induced autophagy flux in HeLa cells by suppressing mTORC1 activity, as revealed by dephosphorylation of the mTORC1 substrates Ulk1, S6K, and 4EBP, as well as by the nuclear translocation of transcriptional factor EB. In addition to non-selective bulk autophagy, TBE also induced aggrephagy, which is defined as autophagy against aggregated proteins. Quercetin is a flavonol found at high levels in TBE. We showed that quercetin induced both non-selective bulk autophagy and aggrephagy. These effects were also observed in Huh-7 cells derived from hepatocytes. Thus, aggrephagy induction by TBE and quercetin may relieve alcoholic hepatitis, which is closely linked to the accumulation of protein aggregations called Mallory–Denk bodies.

## 1. Introduction

Macroautophagy (hereafter referred to simply as autophagy) is an intracellular degradation process that supports cellular homeostasis [1]. The mechanisms that underlie autophagy were initially characterized in yeast studies, but are now understood in mammals as well [2]. As a result, increasing numbers of studies have demonstrated the vital connection between autophagy and a wide variety of human diseases, such as cancer and neurodegenerative diseases [3]. To address these autophagy-related conditions, a number of attempts by the pharmaceutical industry and academia are underway worldwide to artificially regulate autophagy activity in vivo by the use of exogenous compounds [4]. In particular, traditional Chinese medicine has attracted wide attention as a source of potential autophagy modulators [5]. This therapeutic approach has a different foundation than European medicine, and it is broadly accepted, mainly in East Asia. It is based on a long history of prescription and experience, and therefore while its physiological effects are assured to some degree, the underlying molecular mechanisms are largely undetermined. We recently reported that Shigyakusan, a mixture of compounds used in Kampo (Japanese-style traditional Chinese medicine) and a close relative of Shini-san in traditional Chinese medicine, has a suppressive effect on autophagy [6].

Tartary buckwheat (*Fagopyrum tataricum*) is a species of herbaceous plant whose grain is used to prepare flour, noodles, and herbal tea, all mainly in Central and East Asia [7]. Compared to common buckwheat (*Fagopyrum esculentum*)*,* it has a characteristically bitter flavor. In addition to its use in food, the ethanol extract of tartary buckwheat (TBE) is utilized as a component of traditional Chinese medicine [8]. The administration of TBE to mouse brain and liver was shown to reduce antioxidant enzyme levels [8]. It also exerted an antibacterial effect against *Propionibacterium* and *Staphylococci* species [9]. We conducted a pilot survey to determine whether any of the ingredients of Maopu buckwheat liquor, a Chinese liquor containing various Chinese herbal medicine extracts, affected autophagy activity, and found that TBE strongly induced autophagy. Therefore, we performed the present study to elucidate the molecular mechanisms of this property of TBE and its effect on hepatocytes.

## 2. Materials and Methods

### 2.1. Preparation of Tartary Buckwheat Extract

Tartary buckwheat grain powder was provided by Jing Brand Company, Ltd., in Hubei, China. Briefly, tartary buckwheat grain was boiled in 60% (*v*/*v*) ethanol and subjected to centrifugation, and the supernatant was concentrated into powder by vacuum drying centrifugation. The provided power was dissolved in 42% (*v*/*v*) ethanol, heated at 95 °C for 10 min, and then ultra-centrifuged at 100,000× *g* for 60 min at 23 °C. The supernatant, which was stored at −20 °C, was used as TBE.

### 2.2. Antibodies

The following antibodies were used at the indicated dilution ratios for immunoblotting and immunostaining assays: rabbit anti-phospho-ULK1 (Ser757) (6888S, Cell Signaling, MA, USA), 1/1000; mouse anti-ULK1 (C1918, Santa Cruz Biotechnology, TX, USA), 1/1000; rabbit anti-transcriptional factor EB (TFEB) (4240S, Cell Signaling, MA, USA), 1/2000; mouse anti-p70S6 kinase (49D7, 2708S, Cell Signaling, MA, USA), 1/1000; rabbit anti-4E-BP1 (9452S, Cell Signaling, MA, USA), 1/1000; mouse anti-tubulin (T9026, Sigma-Aldrich, St. Louis, MI, USA), 1/10000; rabbit anti-p62 (SQSTM1) (PM045, MBL), 1/1000; rabbit anti-LC3 (PM036, MBL, Tokyo, Japan), 1/1000 (immunoblotting) or 1/500 (immunostaining); HRP-conjugated anti-rabbit IgG secondary antibody (7074S, Cell Signaling, MA, USA), 1/10000; HRP-conjugated anti-mouse IgG secondary antibody (1031-05, Southern Biotech, Birmingham, AL, USA), 1/10000; and Alexa Fluor Plus 488 goat anti-rabbit secondary antibody (A32731, Invitrogen, MA, USA), 1/1000.

### 2.3. Cell Culture

HeLa and Huh7 cells were cultured in Dulbecco’s modified Eagle’s medium (DMEM) (D6429, Sigma-Aldrich, St. Louis, MI, USA) with 10% FBS (F7524, Sigma-Aldrich, St. Louis, MI, USA) and incubated in 5% CO_2_ at 37 °C. HeLa cells stably expressing ULK1-EGFP, GFP-Atg5, GFP-WIPI, tf-LC3, and GFP-TFEB were described previously [6]. For performing LC3 flux assays, 125 nM bafilomycin A1 (023-11641, Wako, Osaka, Japan) was used. For the aggrephagy experiment, cells were treated with 5 μg/mL puromycin (160-23151, Lot PTL1732, Wako, Osaka, Japan) for 4 h and then washed with new medium. Torin-1 (475991, Calbiochem, Darmstadt, Germany) was used at a concentration of 250 nM. Rutin (30319-04, Nacalai tesque, Kyoto, Japan) and was dissolved in 42% ethanol. Quercetin (10005169, Cayman, MI, USA) was first dissolved in DMSO at a concentration of 210 mM and then dissolved with 42% ethanol.

### 2.4. MTT (Methylthialazole Tetrazolium) Assay for Cell Death

Cells were dispensed into 96-well microplates at 5 × 10^4^ cells in 200 µL per well. After overnight culture, cells were treated with the indicated concentrations of TBE for an additional 48 h. Cells were washed in DMEM with 10% FBS and then incubated with 5 µg/mL MTT (345-01821, Dojindo, Tokyo, Japan) in 100 µL medium for 2 h. To lyse MTT-generated crystals, 100 µL of 10% sodium dodecyl sulfate was added after the removal of the MTT medium, and the solution was incubated at 37 °C. Twenty hours later, plates were assessed by spectrophotometry at 570 nm.

### 2.5. Western Blotting

Sample preparation and western blotting were performed, as previously described [6].

### 2.6. Immunofluorescence and Confocal Microscope Observation

Immunofluorescence and confocal microscope observation were performed, as previously described [6]. Images were acquired using a TCS SP8 confocal laser scanning fluorescence microscope (Leica, Wetzlar, Germany) equipped with an objective lens (HC PL APO 63x/1.40 OIL CS2, Leica). The number of fluorescent punctae was determined using ImageJ based on the size threshold specified in each legend. For the analysis of green fluorescent protein (GFP)-TFEB, the fluorescence intensities of certain areas in cytoplasmic and nuclear regions were measured using ImageJ.

## 3. Results

### 3.1. TBE Induces Autophagy

We first performed a pilot investigation using tf-LC3 (tandem fluorescent (GFP-RFP) LC3 protein) in HeLa cells to ascertain whether any of the five ingredients of Maopu buckwheat liquor affect autophagy activity [10]. We discovered that a 42% (*v*/*v*) ethanol extract of TBE induced autophagy. To determine how cell viability was affected by using ethanol as a solvent, HeLa cells were treated with a series of ethanol concentrations from 0 to 10% (*v*/*v*) for 24 h and subjected to an MTT assay. The highest ethanol concentration obtained without affecting cellular viability was 2% (*v*/*v*) (Supplementary Figure S1A). Next, we performed MTT assays with a series of concentrations of TBE dissolved in ethanol, and confirmed that using a concentration of 1500 µg/mL for 6 h did not affect cellular viability (Supplementary Figure S1B). Therefore, we adopted 1500 µg/mL of TBE dissolved in 2% ethanol for further analyses. 

**Figure 1:**
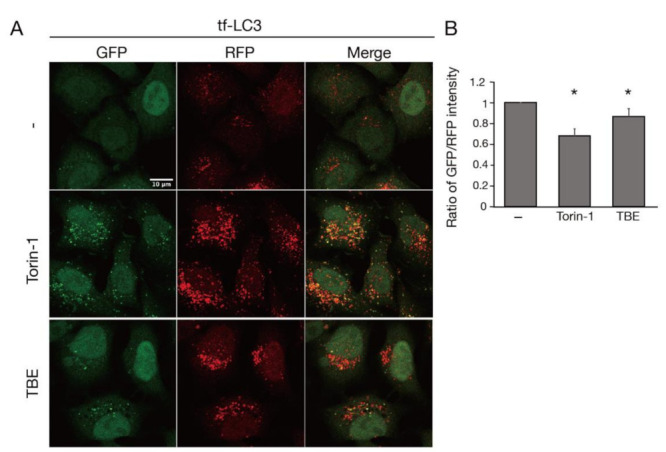
TBE induces autophagy progression. (**A**) Tf-LC3-expressing HeLa cells were treated with or without Torin-1 or TBE for 6 h under nutrient-rich conditions. Bar:10 µm. (**B**) The graph shows the signal intensity ratio of GFP/RFP in each field of view after 6 h. Median: line; upper and lower quartiles: boxes; 1.5 interquartile range: whiskers. * denotes *p* < 0.05 by unpaired two-tailed Student’s *t*-test.

TBE treatment increased the formation of GFP-positive punctae, which represented autophagosomes or isolation membranes (Figure 1A) [11]. RFP-positive punctae were also observed, representing autophagosomes, isolation membranes, or autolysosomes (Figure 1A) [12]. The decrease in the ratio of the GFP/RFP signal intensity indicated that autophagosomes or isolation membranes had disintegrated during autophagy. Torin-1, a well-established inducer of autophagy, decreased the ratio of the GFP/RFP signal intensity (Figure 1B) [13]. Likewise, TBE decreased the GFP/RFP signal intensity in comparison to vehicle control (Figure 1B).

**Figure 2:**
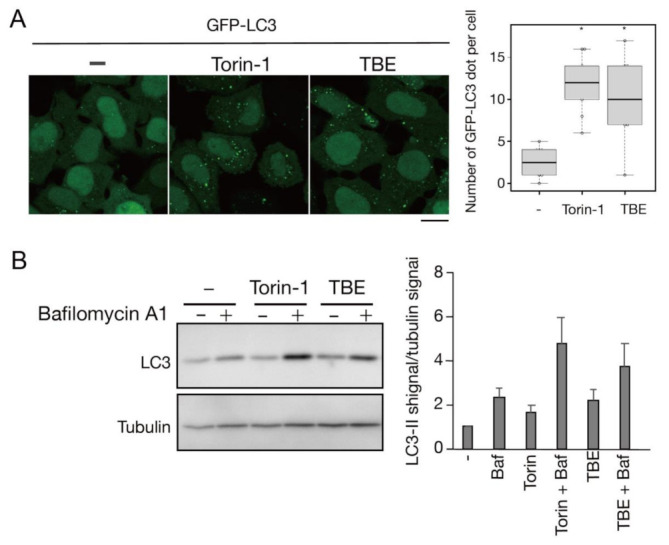
TBE induces autophagy flux under nutrient-rich conditions. (**A**) GFP-LC3-expressing HeLa cells were treated with Torin-1 or TBE for 6 h under nutrient-rich conditions. The number of GFP punctae per cell was counted, with over 20 cells in each sample. Median: line; upper and lower quartiles: boxes; 1.5 interquartile range: whiskers. Bar: 10 µm (**B**) HeLa cells were treated with Torin-1 or TBE with or without bafilomycin A1 for 6 h. The lysates were assessed by Western blotting with anti-LC3 antibody. The graph shows the average and standard deviation of the ratio of the LC3 signal to the tubulin signal from three independent experiments. * denotes *p* < 0.05 by unpaired two-tailed Student’s *t*-test.

We next assessed TBE-induced autophagy activity using a flux assay based on Western blotting of the autophagy marker LC3 [14]. Degradation of the LC3-II form, which is covalently conjugated to phosphatidylethanolamine, occurs upon fusion of autophagosomes with lysosomes. The extent of autophagy induction can be assessed by inhibiting this degradation by treatment with bafilomycin A1, a lysosomal acidification inhibitor. HeLa cells were treated with TBE for 6 h with or without bafilomycin A1, along with vehicle only (DMEM) or Torin-1 as controls (Figure 2). The results showed that LC3-II accumulated in samples treated with either TBE or Torin-1, indicating the induction of autophagy.

### 3.2. TBE Induces Autophagosome Formation

**Figure 3:**
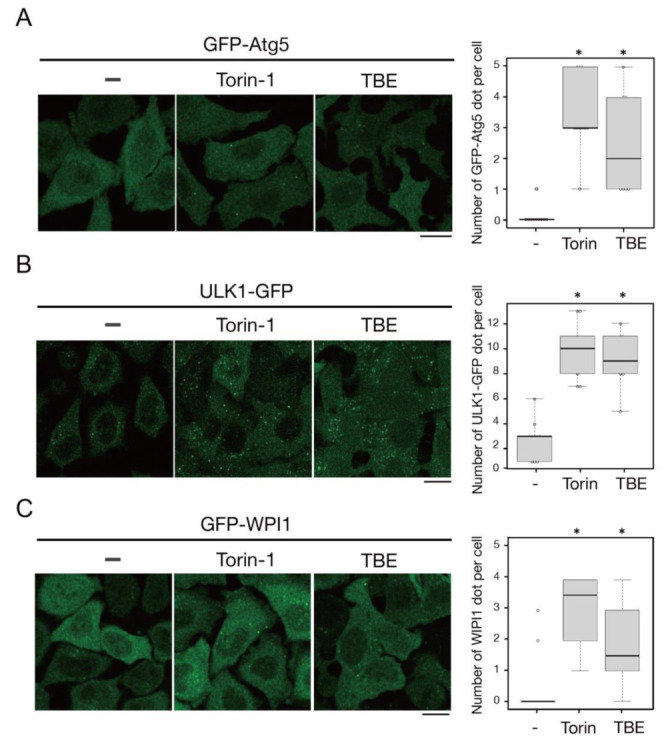
TBE induces autophagosome formation. HeLa cells expressing GFP-Atg5 (**A**), ULK1-EGFP (**B**), or GFP-WIPI1 (**C**) were treated with Torin-1 or TBE for 4 h. The cells were observed under a confocal microscope. The number of GFP punctae over 0.56 µm in diameter was counted per cell, with over 20 cells in each sample. Median: line; upper and lower quartiles: boxes; 1.5 interquartile range: whiskers. Bar: 10 µm. * denotes *p* < 0.05 by unpaired two-tailed Student’s *t*-test.

We next examined whether TBE affected autophagosome formation. Atg5 is a protein involved in the autophagosome formation process; it associates with isolation membranes and is then released from these membranes after autophagosome formation is complete [15]. Therefore, Atg5-positive structures represent isolation membranes/phagophores. TBE treatment increased GFP-Atg5-positive puncta structures in HeLa cells even under nutrient-rich conditions, indicating the formation of autophagosomes (Figure 3A). ULK1 is a protein kinase that comprises a protein complex along with FIP200/RB1CC1, Atg101, and Atg13; this complex constitutes a scaffold for autophagosome formation [16]. TBE treatment induced ULK1-EGFP puncta signals under nutrient-rich conditions (Figure 3B). WIPI1 is another core protein that is recruited to isolation membranes/phagophores and autophagosomes as a result of its binding affinity for phosphatidylinositol 3-phosphate [17]. The number of GFP-WIPI1 punctae was increased by TBE treatment (Figure 3C). Collectively, these data indicate that TBE induces autophagy in the step before autophagosome formation.

### 3.3. TBE Enhances Dephosphorylation of mTORC1 Substrates

**Figure 4:**
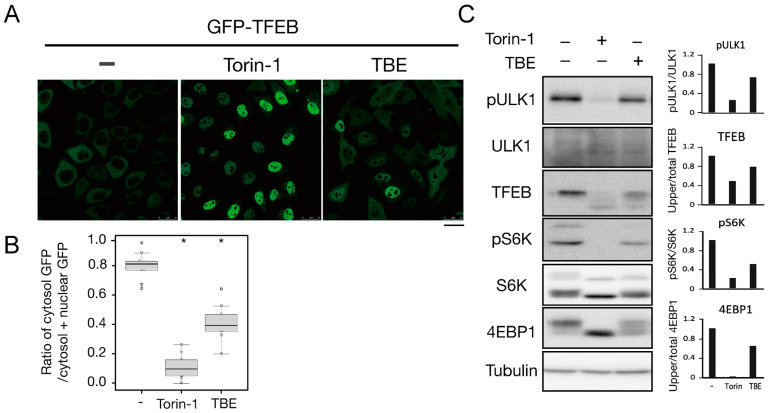
TBE enhances dephosphorylation of mTORC1 substrates. (**A**) GFP-TFEB-expressing HeLa cells were treated with or without Torin-1 or TBE for 4 h and observed under a confocal microscope. Bar: 25 µm. (**B**) The graph shows the ratio of GFP intensity in a 7 µm^2^ of the cytosol to the sum of GFP intensities in certain areas of the cytoplasm and nucleus. Median: line; upper and lower quartiles: boxes; 1.5 interquartile range: whiskers. * denotes *p* < 0.05 by unpaired two-tailed Student’s *t*-test. (**C**) HeLa cells were treated with or without Torin-1 or TBE for 4 h and subjected to Western blotting using anti-phospho-ULK1 (Ser757), anti-ULK, anti-TFEB, anti-phospho-S6K, anti-p70 kinase, anti-4E-BP1, and anti-tubulin antibodies. The graph shows the quantification of each band intensity.

To investigate how TBE induced autophagy, we focused on the protein kinase mTORC1, which negatively regulates autophagy induction by alternating the phosphorylation level of proteins involved in the autophagy regulation process [13]. Transcriptional factor EB (TFEB) is a mTORC1 kinase substrate and an important regulator of autophagy/lysosome protein biogenesis [18]. Under nutrient-rich conditions, active mTORC1 phosphorylated TFEB and retained TFEB in the cytoplasm, whereas dephosphorylated TFEB under starvation conditions translocated into the nucleus. However, when the cells were treated with TBE, GFP-TFEB was localized in the nucleus even under nutrient-rich conditions (Figure 4A,B).

We next examined the phosphorylation levels of mTORC1 substrates by Western blotting. The band size of TFEB was shifted down in response to Torin-1, indicating that TFEB was dephosphorylated (Figure 4C). TFEB was also shifted when the cells were treated with TBE (Figure 4C).

ULK1 protein kinase was another mTORC1 kinase substrate. The serine-757 site of ULK1 was phosphorylated by active mTORC1 to suppress autophagy, while treatment with the mTORC1 inhibitor Torin-1 led to ULK1 dephosphorylation at this serine site [19]. TBE treatment also resulted in dephosphorylation of this site (Figure 4C). The other major mTORC1 substrates, the ribosomal protein S6 kinase (S6K) and the translation initiation factor 4E-binding protein (4E-BP) [20], were also dephosphorylated in the presence of TBE (Figure 4C). Collectively, these results show that TBE suppresses mTORC1 kinase activity.

### 3.4. TBE Induces Autophagy against Protein Aggregation

**Figure 5:**
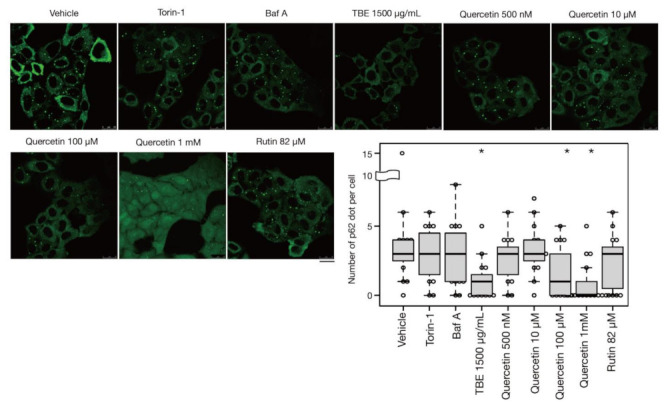
TBE and quercetin enhance the induction of aggrephagy. HeLa cells were treated with puromycin for 4 h, Figure 1. Bafilomycin A1, TBE, quercetin, or rutin at the indicated concentrations for 1 h. p62 was detected by immunostaining. The graph shows the number of p62 dots over 1.4 µm in diameter per HeLa cell. Bar: 25 µm. Median: line; upper and lower quartiles: boxes; 1.5 interquartile range: whiskers. * denotes *p* < 0.05 by unpaired two-tailed Student’s *t*-test.

In addition to bulk autophagy, which degrades nonselective cytosolic components, a wide range of specific targets are also subjected to autophagy; in this case, the process is known as selective autophagy [21]. Among these targets are aggregated proteins which are engulfed by autophagosomes and eventually degraded by a process called aggrephagy, one form of selective autophagy. In a previous study, protein aggregates were artificially induced by halting translation with puromycin treatment [22]. P62/SQSTM-1, an autophagy-related adaptor protein, is localized with protein aggregations and can be utilized as a protein aggregation marker [23]. In this study, p62-positive punctae formed after treatment with puromycin for 4 h, representing protein aggregates (Figure 5). Eighteen hours after puromycin was washed out, most p62-positive punctae disappeared, although these punctae persisted in autophagy-defective HeLa cells [22] [Ikari et al., manuscript in preparation], indicating that their removal was dependent on aggrephagy. Torin-1 treatment accelerated this process and resulted in a reduced number of p62-positive punctae, but the effect was moderate after just a 1-h washout (Figure 5). However, TBE treatment enhanced the clearance of p62 punctae even within 1 hr (Figure 5), indicating that TBE induces aggrephagy.

### 3.5. Quercetin Is Responsible for the TBE Effect on Autophagy Induction

**Figure 6:**
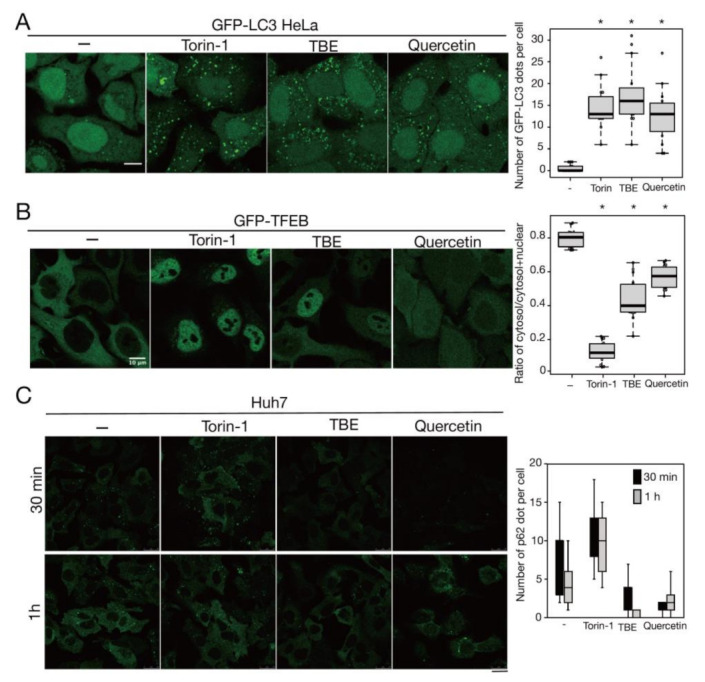
Quercetin enhances autophagy. (**A**) GFP-LC3-expressing HeLa cells were treated with or without Torin-1, TBE, or quercetin for 4 h under nutrient-rich conditions. The graph shows the number of GFP-LC3 punctae over 1.3 µm in diameter per cell. (**B**) GFP-TFEB-expressing HeLa cells were treated with or without Torin-1, TBE, or quercetin for 4 h under nutrient-rich conditions. The graph shows the ratio of GFP intensity in a certain area of the cytosol to the sum of GFP intensities in certain areas of the cytoplasm and nucleus. (**C**) Huh7 cells were treated with puromycin for 4 h, and further cultured with or without Torin-1, TBE, or quercetin for 1 h after washing out puromycin. Intracellular p62 was detected by immunostaining. The graph shows the number of p62 dots over 2.5 µm in diameter per cell. Median: line; upper and lower quartiles: boxes; 1.5 interquartile range: whiskers. Bar: 10 µm for (**A**,**B**); 25 µm for (**C**). * denotes *p* < 0.05 by unpaired two-tailed Student’s *t*-test.

Rutin is a flavonoid that occurs at much higher concentrations in tartary buckwheat than in common buckwheat. We examined whether rutin could induce autophagy, but it did not do so even at a high concentration (Figure 5). Rutin consists of the flavonol quercetin and the disaccharide rutinose. A previous report showed that TBE includes a highly active rutin-degrading enzym, and, as a result, rutin is promptly degraded into quercetin in aqueous extraction [24,25]. We therefore examined whether quercetin affected autophagy. The TBE batch used in this study comprised 2.8% (*w*/*w*) of quercetin according to HPLC analysis (data not shown), which is equivalent to 130 µM in 1500 µg/mL of TBE. We treated HeLa cells to determine how aggrephagy was affected by a series of quercetin concentrations. Aggrephagy was promoted by 100 µM of quercetin, but not by 25 or 50 µM (Figure 5). We further examined the effects of quercetin on autophagy using GFP-LC3-expressing HeLa cells. When quercetin was added, the number of GFP-LC3 punctae was higher compared to vehicle control (Figure 6A). We also examined the effect of quercetin treatment on mTORC1 activity. When HeLa cells expressing GFP-TFEB were treated with quercetin, GFP-TFEB showed nuclear translocation similar to that seen following treatment with Torin-1 or TBE (Figure 6B).

### 3.6. TBE and Quercetin Promote Aggrephagy in Liver Cells

Mallory–Denk bodies are hepatocytic protein aggregates that are closely linked to the pathogenesis of alcoholic hepatitis [26]. A previous study reported that p62 aggregates accumulated in the liver of autophagy-deficient mice [27]. To study the effects of TBE and quercetin in the liver, we performed the same experiments described above using Huh7 cells, which are derived from hepatocytes. Like HeLa cells, Huh7 cells demonstrated aggrephagy induction by TBE and quercetin (Figure 6C).

## 4. Discussion

In this study, we showed that TBE treatment induced autophagy by suppressing mTORC1 activity, and that quercetin was the ingredient that exerted this effect. We further revealed that TBE and quercetin promoted not only bulk autophagy, but also aggrephagy against aggregated proteins.

The initial phase of this project investigated the effects on autophagy of five ingredients in Maopu buckwheat liquor, and we eventually succeeded in identifying quercetin as the ingredient with potent aggrephagy-inducing ability. It remains possible that compounds in TBE other than quercetin also contribute to this effect. However, quercetin may be the primary ingredient capable of inducing autophagy on its own. Previous studies have reported that quercetin is associated with autophagy in several contexts. First, quercetin treatment induced autophagy in gastric cancer cells, causing a pronounced pro-apoptotic effect [28]. Subsequently, Klappan et al. reported that quercetin possesses a proteasome inhibitory effect that leads to mTORC1 inhibition [29]. However, in contrast with our findings, their study reported that quercetin treatment induced protein aggregation, mostly inside the nucleus [29]. Induction of autophagy by quercetin has been observed in several tissues and diseases, including diabetic nephropathy [30], oocytes from aged mice [31], intervertebral disc degeneration [32], myelodysplastic bone marrow [33], and human retinal pigment epithelial cells [34], while one study reported that quercetin attenuated autophagy in a rat model of traumatic brain injury [35]. Regardless of these previous studies, here we performed a comprehensive set of autophagy assays that unquestionably proved the effect of quercetin on autophagy for the first time [36]. As suggested by our study and the aforementioned reports, these effects could be mediated by mTORC1 suppression. The underlying molecular mechanism is still to be determined, but a recent report that quercetin directly binds to mTORC1 has interesting implications [37].

Both TBE and quercetin induce bulk autophagy and aggrephagy. TBE and quercetin are less effective at suppressing mTORC1 than Troin-1. Nevertheless, TBE and quercetin induce aggrephagy at an even earlier time (1 hr) than the point at which Torin-1 exerted its limited effect on aggrephagy. Therefore, we speculate that a pathway other than that involving mTORC1 is affected by quercetin in aggrephagy, although the identity of this additional pathway is still to be determined. In the liver, the pathogenesis of alcoholic hepatitis is closely linked to the accumulation of protein aggregates called Mallory–Denk bodies [26], and the clearance of these aggregates could potentially alleviate hepatitis. In addition, protein aggregates in the liver provoke tumorigenesis [38], and aggrephagy is capable of clearing these proteins [27]. Quercetin is linked to alcohol-induced liver injury because it enhances autophagy against mitochondria (mitophagy) [39] and lipid droplets (lipophagy) [40]. Combined with these effects, the ability of TBE to enhance aggrephagy could potentially prevent these diseases. Future research to clarify this possibility is needed, especially using animal models.

## Data Availability

The data presented in this study are openly available in FigShare at DOI [10.6084/m9.figshare.15057939].

## Supplementary Materials

The following are available online at https://www.mdpi.com/article/10.3390/antiox10081217/s1, Figure S1: The effects of ethanol and TBE on cell viability.
