# Supplementary material for: Quercetin in Tartary Buckwheat Induces Autophagy against Protein Aggregations
Source: Antioxidants (Basel). 2021 Jul 29;10(8):1217. doi: 10.3390/antiox10081217 (PMC8388858; doi:10.3390/antiox10081217)
Supplement: Supplementary file 1 [file antioxidants-10-01217-s001.zip › antioxidants-1272525-supplementary.pdf]

Supplementary figure 1

A

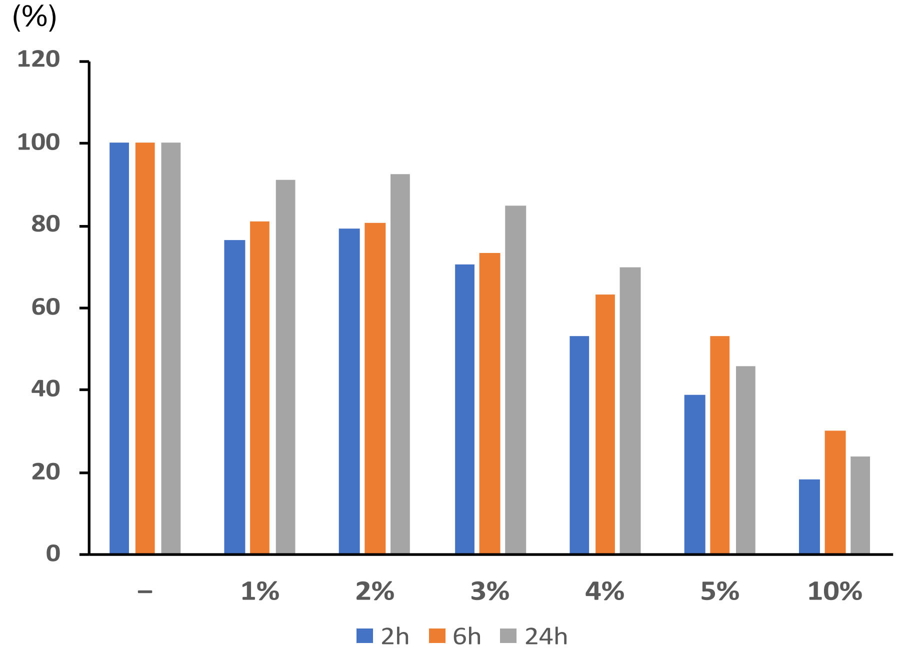

B

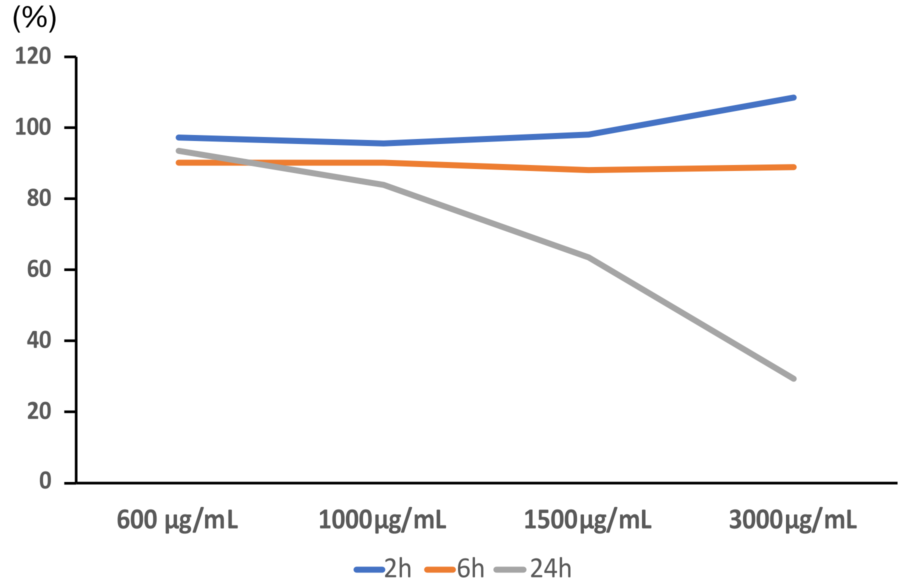

Supplementary Fig 1. The effects of ethanol and TBE on cell viability. (A) HeLa cells were treated with ethanol at 0 (-), 1, 2, 3, 4, 5, and 10% (v/v) for the indicated periods, and MTT assays were performed. Relative survival rates compared to 0% are presented. (**B**) HeLa cells were treated with TBE at final concentrations of 600, 1000, 1500, and 3000 μg/mL for the indicated periods under nutrient-rich conditions, and MTT assays were performed. Relative survival rates compared to time 0 (Control) are presented.
